# Pre-operative ultrasound mapping before arteriovenous fistula formation: an updated systematic review and meta-analysis

**DOI:** 10.1007/s40620-023-01814-6

**Published:** 2023-12-22

**Authors:** David-Dimitris Chlorogiannis, Stelios-Elion Bousi, Marinos Zachiotis, Anargyros Chlorogiannis, Ioannis Kyriakoulis, Ioannis Bellos

**Affiliations:** 1 Department of Research Methodology and Biostatistics, Aristotle University of Thessaloniki, 541 24 Thessaloniki, Greece; 2 First Department of Surgery, National and Kapodistrian University of Athens, Laiko General Hospital, National and Kapodistrian University of Athens, 11527 Athens, Greece; 3 Department of Health Economics, Policy and Management, Karolinska Institutet, Stockholm, Sweden; 4 Department of Internal Medicine, School of Health Sciences, University of Thessaly, Larissa, Greece; 5 Department of Nephrology and Renal Transplantation, Laiko General Hospital, National and Kapodistrian University of Athens, 11527 Athens, Greece; 6 Department of Hygiene, Epidemiology and Medical Statistics, Medical School, National and Kapodistrian University of Athens, 17, Agiou Thoma Str., 11527 Athens, Greece

**Keywords:** Ultrasound, Doppler, Mapping, Arteriovenous fistula, Dialysis

## Abstract

**Background:**

Arteriovenous fistula represents the preferred vascular access for patients with kidney failure requiring hemodialysis. Surgeons have traditionally used physical examination to identify the most suitable vessels. This meta-analysis aims to evaluate whether ultrasound mapping should be routinely performed before arteriovenous fistula creation.

**Methods:**

Medline, Scopus, Web of Science and CENTRAL were systematically searched from inception to November 1, 2022. Randomized controlled trials and cohort studies comparing routine ultrasound mapping to physical examination in terms of arteriovenous fistula patency were included. Meta-analysis was performed by fitting random-effects models. The study protocol has been prospectively registered in PROSPERO (CRD42023402390).

**Results:**

Overall, 18 studies were included, comprising 3655 participants. Routine pre-operative ultrasound mapping was associated with significantly lower rates of primary arteriovenous fistula failure (Risk Ratio-RR: 0.56, 95% confidence intervals-CI: 0.37–0.84, low certainty). A significant outcome was observed by separately pooling randomized controlled trials (RR: 0.37, 95% CI: 0.25–0.54). Routine ultrasound mapping was also associated with significantly higher rates of 1-year primary arteriovenous fistula patency (RR: 1.33, 95% CI: 1.19–1.47, moderate certainty). This effect remained significant in the analysis of randomized controlled trials (RR: 1.26, 95% CI: 1.02–1.56).

**Conclusions:**

Implementing routine pre-operative ultrasound mapping of vessels is associated with significantly better outcomes in terms of early arteriovenous fistula failure and primary patency rates at 12 months. Further research should confirm the long-term benefits of routine ultrasound examination and evaluate its cost-effectiveness in different populations.

**Graphical Abstract:**

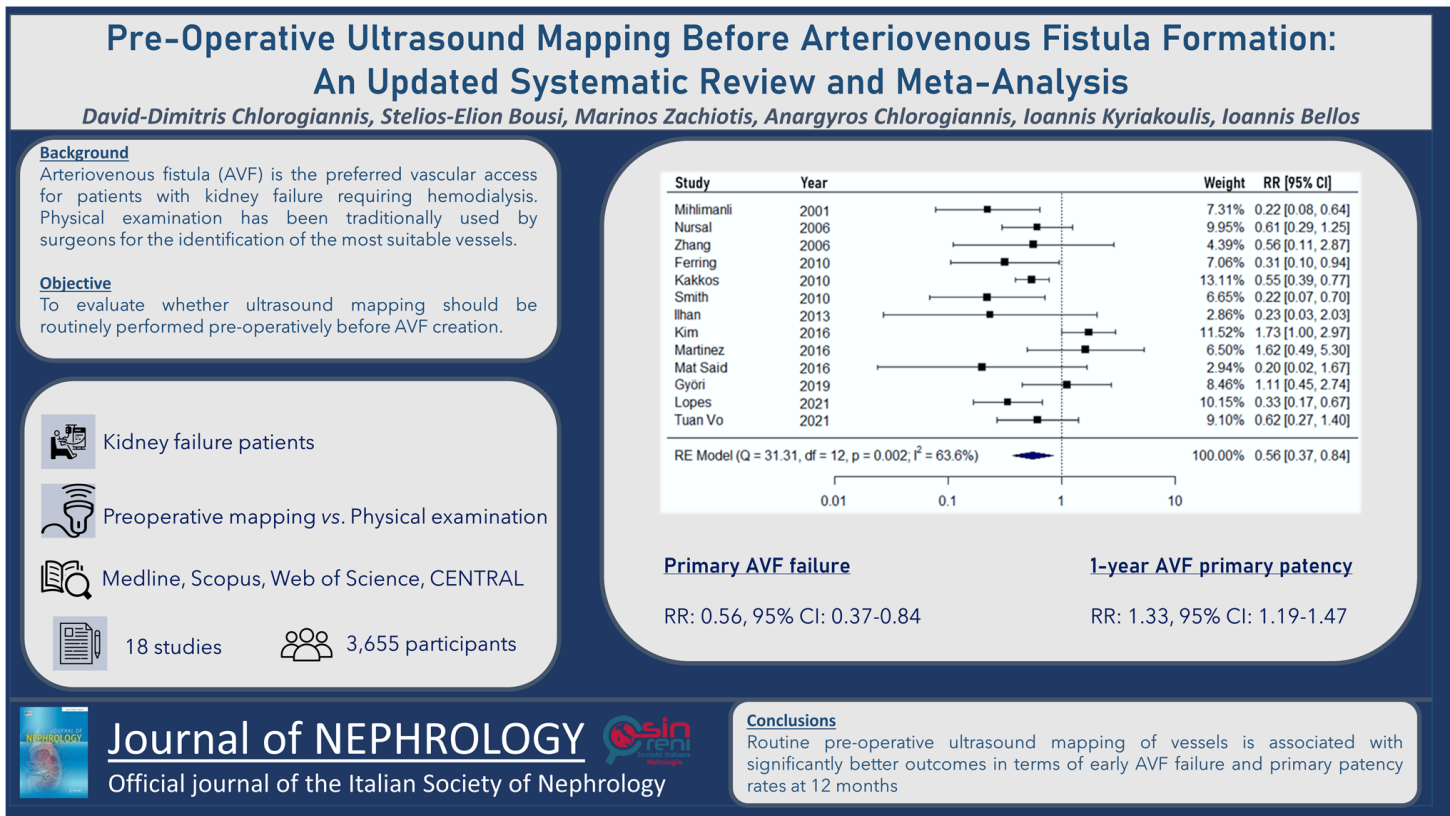

**Supplementary Information:**

The online version contains supplementary material available at 10.1007/s40620-023-01814-6.

## Introduction

Kidney failure on renal replacement therapy affects more than 4 million people worldwide and hemodialysis represents the main form of kidney replacement therapy [1]. The creation of an arteriovenous fistula is recommended as the primary vascular access option for the initiation of hemodialysis [2]. The successful creation of an arteriovenous fistula depends on several factors, with the incidence of 1-year primary failure rate being reported as high as 34% [3]. In addition, patients may require multiple salvage percutaneous or open interventions or even repeat vascular access procedures, which exacerbates cost-effectiveness and reduces the quality of life.

To identify a suitable candidate vessel and increase patency rates, preoperative ultrasound mapping of the vessels of the upper limbs versus conventional physical examination alone is recommended by the Spanish Clinical Guidelines for Vascular Access [2]. Rather than routine vascular mapping, the National Kidney Foundation (The Kidney Disease Outcomes Quality Initiative (KDOQI)) suggests selective preoperative ultrasound evaluation in patients at high risk of AV access failure [4]. Sonography allows for more precise measurement of internal vessel diameters, with a minimum of 2 mm of arteries and veins typically recommended. At the same time, it also provides valuable information concerning internal vascular lesions, which cannot be detected by physical examination alone [5, 6]. However, the level of evidence of such guidelines relies on randomized controlled studies which include small sample size, while there is a paucity of data concerning the value of preoperative ultrasound in arteriovenous fistula long-term patency, since their quantitative synthesis did not include one-year patency.

This systematic review and meta-analysis aims to analyze the updated clinical evidence concerning the value of routine preoperative sonographic mapping for arteriovenous fistula formation and its long-term primary failure rates in comparison to physical examination or selective ultrasound alone.

## Materials and methods

### Study design

The meta-analysis was designed following the PRISMA (Preferred Reporting Items for Systematic reviews and Meta-Analyses) guidelines [7]. The study protocol has been prospectively registered in PROSPERO (CRD42023402390). Ethical approval was omitted since no new patients were involved in this study.

### Eligibility criteria

The population of the meta-analysis consisted of adult patients with kidney failure undergoing first-time arteriovenous fistula creation for dialysis initiation. Routine pre-operative Doppler ultrasound evaluation was compared to physical examination with or without selective ultrasound. The outcomes of interest were primary arteriovenous fistula failure (immediate arteriovenous fistula failure, early thrombosis or maturation failure) and primary patency at 12 months after surgery (functional arteriovenous fistula for hemodialysis without the need for intervention). Cohort studies (both prospective and retrospective) and randomized controlled trials (RCTs) were deemed eligible. Case–control, cross-sectional, descriptive, animal and in vitro studies were excluded.

### Literature search

The systematic literature search was conducted on Medline (via PubMed), Scopus, Web of Science, CENTRAL (Cochrane Central Register of Controlled Trials); the full reference lists of the retrieved studies were also searched to identify additional articles (“snowball” method [8]). Databases were searched from inception until November 1, 2022. The search was based on a combination of keywords and MeSH (Medical Subject Headings) terms. The main applied algorithm was the following: ((venous mapping) OR (vessel mapping) OR (ultrasound) OR (pocus) OR (sonography) OR (point-of-care-ultrasound) OR (Doppler) OR (“Ultrasonography”[Mesh])) AND ((dialysis) OR (hemodialysis) OR (“Renal Dialysis”[Mesh])) AND ((fistula) OR (arteriovenous fistula) OR (AVF vascular access) OR (“Fistula”[Mesh])). No language restrictions were applied. The search was performed independently by two researchers and any discrepancies were resolved by consensus.

### Study selection

The study selection followed three consecutive steps. Firstly, the titles and abstracts of all electronic records were screened for potential eligibility. Subsequently, the articles considered as possibly eligible were retrieved as full-texts. Then, any studies that did not report the outcomes of interest or fulfilled any of the exclusion criteria were excluded. The selection of studies was independently performed by two researchers, resolving any potential disagreement with the consensus of all authors.

### Data extraction

Dedicated forms were used for extraction of the following data from the included studies: year of publication, country, study period, design, sample size, eligibility criteria, participants’ median age and sex, as well as the percentage of patients with hypertension and diabetes mellitus. Information regarding the outcomes of interest (early arteriovenous fistula failure and 12-month primary patency) was also collected. Two authors collected data, resolving any potential discrepancies after a discussion with a third author.

### Quality assessment

The risk of bias of the included RCTs was evaluated using the RoB-2 tool [9], assessing the following domains: randomization, deviations from intended interventions, missing outcome data, measurement of outcomes and selection of the reported results. Risk of bias evaluation of cohort studies was performed using the ROBINS-I tool [10], which takes into account the following domains: confounding, selection of participants classification of interventions, deviations from intended interventions, missing data, measurement of outcomes and selection of the reported results. The risk of bias was judged as low, moderate, serious or critical. The process of quality assessment was conducted independently by two researchers, blinded to each other, and any discrepancy was resolved through the consensus of all authors.

### Statistical analysis

Data analysis was performed in R-4.0.5 (package “metafor” [11]). Statistical significance was defined by a two-sided *p-value* threshold of 0.05. Random-effects models using the maximum likelihood method were fitted to provide estimates of risk ratio (RR) along with their 95% confidence intervals (CI). The degree of statistical heterogeneity was quantified by the inconsistency index (*I*^*2*^), with values > 50% denoting remarkable heterogeneity [12]. Additionally, the 95% predictive intervals were calculated to provide estimates of the effects to be expected by future studies [13]. Subgroup analysis was performed by categorizing studies based on study location, year of publication (≤ 2010 vs. > 2010), sample size (≤ 150 vs. > 150 patients), study design (cohort vs. RCT) and risk of bias. Publication bias was assessed by the visual inspection of funnel plots, while the trim-fill method was applied to account for potentially missing studies after statistical imputation [14]. The Egger’s regression test was used to evaluate funnel plot asymmetry in the case of at least 10 included studies [15].

### Certainty of evidence

The GRADE approach was used to evaluate the certainty of evidence per outcome. Specifically, the certainty of evidence was judged as very low, low, moderate or high by taking into account the following domains: study limitations, inconsistency, indirectness, imprecision and publication bias [16].

## Results

### Study selection

**Fig. 1:**
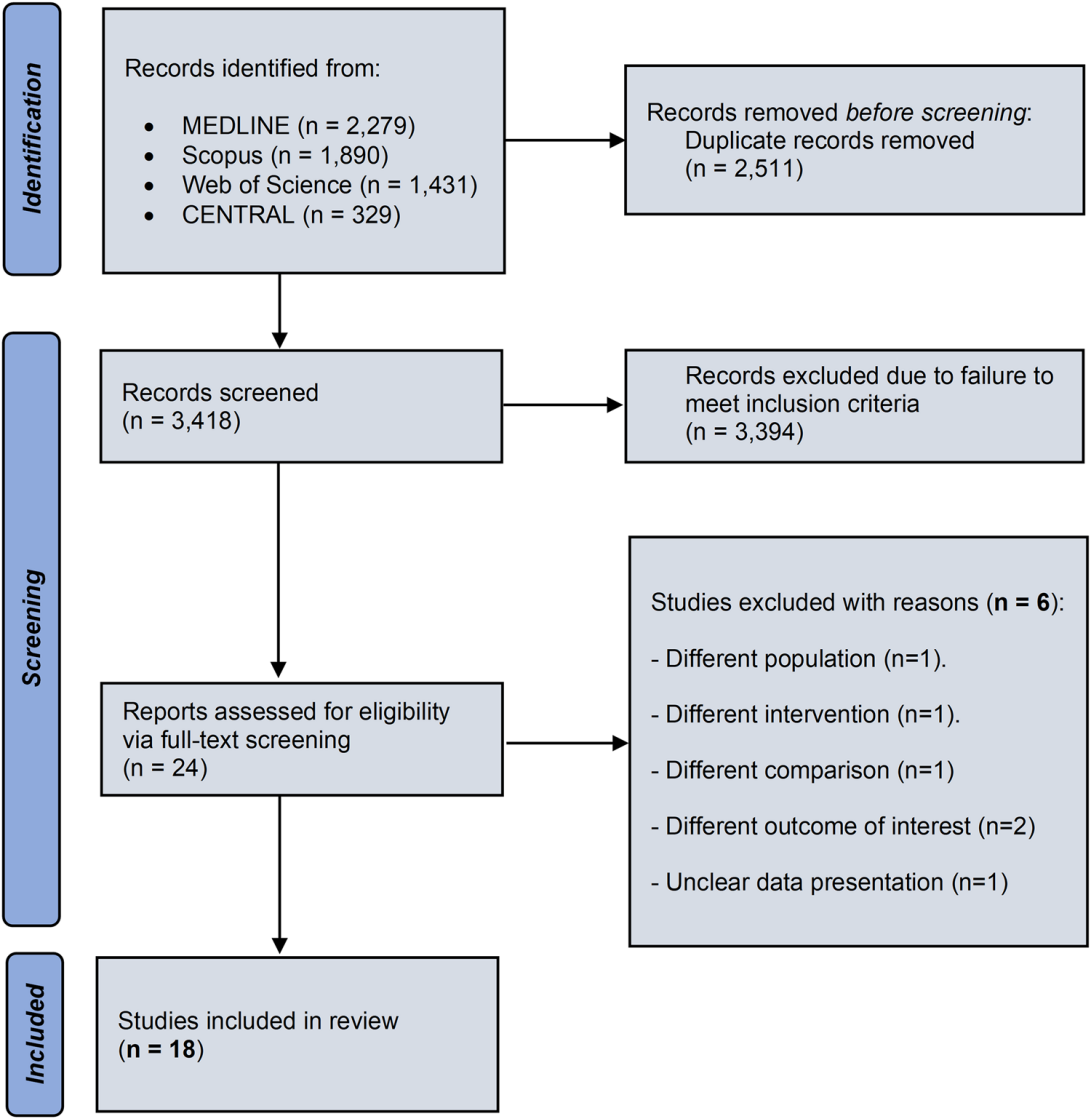
Search plot diagram

The study selection process is schematically depicted in Fig. 1. The literature search resulted in a total of 5929 records. After the removal of duplicates, 3418 articles were screened for eligibility and 24 of them were retrieved in full-text form. Subsequently, 6 studies were excluded due to the following reasons: different population (*n* = 1) [17], different intervention (*n* = 1) [18], different comparison (*n* = 1) [19], different outcome of interest (*n* = 2) [20, 21] and unclear data presentation (*n* = 1)[22]. As a result, a total of 18 studies were included in the meta-analysis, comprising 3,655 participants [23–40]. In 1693 of them, routine pre-operative ultrasound examination was performed, while in 1962 individuals arteriovenous fistula formation was based on physical examination.

### Included studies

The methodological characteristics of the included studies are presented in Table 1. Six studies were RCTs [23, 36–40], 3 were prospective cohorts [25, 29, 34] and 9 studies followed a retrospective cohort design. [24, 26–28, 30–33, 35] Nine studies were conducted in Asia, 6 in Europe, 2 in North America and 1 in South America (Table 1). In the vast majority of studies, both arterial and venous ultrasound mapping were performed. The risk of bias in RCTs was judged to be moderate due to uncertainty and heterogeneity in the domain of outcomes measurement (Suppl. Figure 1). Regarding observational studies, the overall risk of bias was evaluated to be low in 1 study, moderate in 8 studies and serious in 3 studies. No study was judged to be at critical risk of bias (Suppl. Table 1). The main source of bias concerns was the domain of confounding reflecting the differences in the baseline characteristics of participants. In addition, bias in the domain of participant selection was recognized in studies where the non-randomized design was assessed to significantly affect the allocation of patients to routine or selective ultrasound mapping.

**Table 1:** Methodological characteristics of the included studies

Study	Country	Sample size	Study design	Exclusion criteria	Arterial/venous mapping	Median age (years) †	Male sex (%) †	Hypertension (%) †	Diabetes mellitus (%) †	Risk of bias
2001; Mihmanli	Turkey	124	RCT	Age > 74 years, previous AVF	Both	NR	58.3 vs. 51.9	NR	NR	Moderate
2006; Nursal	Turkey	66	RCT	External venous diameter < 1 mm, visible vein length < 5 cm, inadequate pulsatile force, arm edema	Both	56.5 vs. 56.5	51.4 vs. 54.3	94.1 vs. 82.9	44.1 vs. 37.1	Moderate
2006; Zhang	China	66	RCT	NR	Both	NR	NR	NR	NR	Moderate
2007; Karakayali	Turkey	280	Retrospective cohort	NR	Both	52.8 vs. 46.9*	54.6 vs. 56.3	NR	35 vs. 35.1	Moderate
2010; Ferring	UK	208	RCT	> 1 previous AVF, previous AVG	Both	69 vs. 67	61.6 vs. 66.0	78.4 vs. 75.2	43.2 vs. 34.3	Moderate
2010; Kakkos	USA	467	Prospective cohort	NR	Both	62 vs. 64	52.7 vs. 59.3	NR	50 vs. 45	Serious
2010; Smith	UK	77	RCT	Previous vascular intervention on target limb, thrombophilia	Both	64.3 vs. 65.3	NR	63.8 vs. 57.4	31.9 vs. 31.9	Moderate
2013; Ilhan	Turkey	118	Retrospective cohort	Central vein stenosis, outflow vein occlusion, arm edema	Both	56.4 vs. 54.5	57.1 vs. 57.9	50.7 vs. 44.7	38 vs. 34.2	Moderate
2016; Kim	South Korea	469	Retrospective cohort	Central vein stenosis, outflow vein occlusion	Both	55.6 vs. 55.8	52.5 vs. 65.2*	85.6 vs. 90.1	60.2 vs. 43.9*	Moderate
2016; Martinez	Spain	81	Retrospective cohort	Previous AVF in same extremity	Both	68.3 vs. 64.8	67 vs. 60	90 vs. 86	64 vs. 47	Moderate
2016; Mat Said	Malaysia	158	Prospective cohort	Previous AVG, AVF re-intervention	Both	52.9 vs. 52.5	45.5 vs. 57	91.1 vs. 87.3	59.5 vs. 55.7	Moderate
2016; Giannikouris	Italy	102	Retrospective cohort	Non-diabetic patients, previous AVF/AVG	Both	65 vs. 69	64.7 vs. 70.6	NR	NR	Moderate
2018; Hossain	Canada	316	Retrospective cohort	Loop AVG, AVF for plasmapheresis	Venous	59 vs. 60	73 vs. 71	88 vs. 90	48 vs. 57	Moderate
2018; Kim	South Korea	250	Retrospective cohort	Arterial stenosis/occlusion	Both	56.3 vs. 56.9	62.7 vs. 66.1	81 vs. 69.4*	62.7 vs. 54.8	Moderate
2019; Györi	Austria	331	Retrospective cohort	NR	Both	60.7 vs. 58.7	70.2 vs. 58.1*	65.8 vs. 73.7	22.8 vs. 31.3	Serious
2019; Torres	Spain	178	Prospective cohort	Unavailable ultrasound, AVF site indicated by clinical events	Both	64 vs. 68.4*	63 vs. 61	92 vs. 92	55 vs. 63	Serious
2021; Lopes	Brazil	206	RCT	Central vein stenosis, arterial stenosis/occlusion	Both	56 vs. 57.5	65 vs. 71	46.5 vs. 53.5	40.4 vs. 39.5	Moderate
2022; Tuan Vo	Vietnam	158	Retrospective cohort	No start of hemodialysis	Both	54.1 vs. 55.4	43 vs. 32.9	88.6 vs. 88.6	36.7 vs. 29.1	Low

*NR* not reported, *RCT* randomized controlled trial, *AVF* arteriovenous fistula, *AVG* arteriovenous graft

^†^Comparisons of routine ultrasound group vs. physical examination group

*p value < 0.05

### Primary arteriovenous fistula failure

**Fig. 2:**
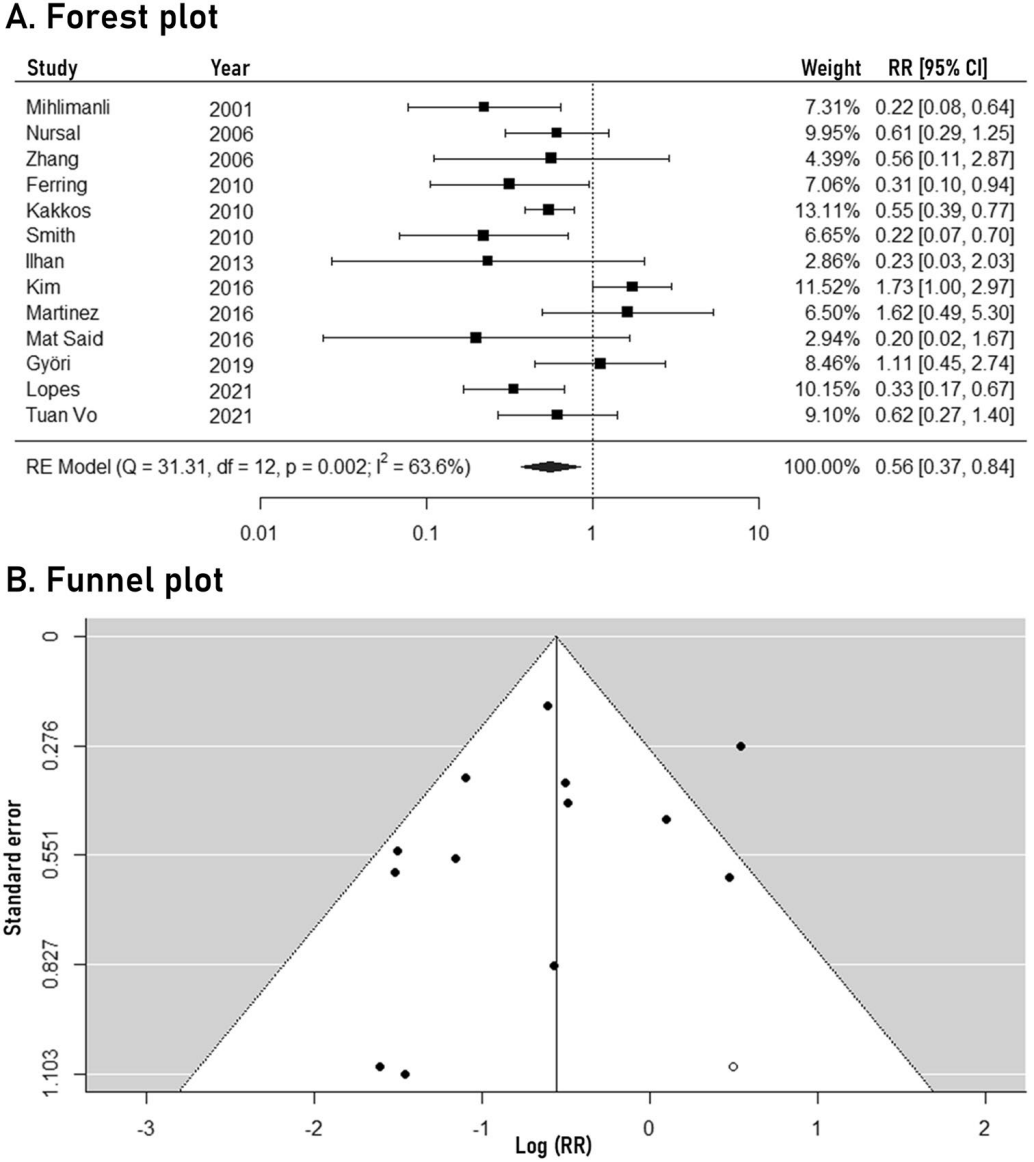
Forest plot (**A**) and funnel plot (**B**) of the pre-operative ultrasound mapping effects on primary arteriovenous fistula failure

The meta-analysis outcomes regarding primary arteriovenous fistula failure are depicted in Fig. 2. Routine pre-operative ultrasound mapping was associated with significantly lower rates of primary arteriovenous fistula failure (RR: 0.56, 95% CI: 0.37 to 0.84, 13 studies). Moderate statistical heterogeneity was noted (*I*^*2*^: 63.6%), while the 95% predictive intervals ranged from 0.18 to 1.75. No significant funnel plot asymmetry was observed (Egger’s *p-value*: 0.181). The trim-fill method identified one potentially missing study, not affecting the significance of the pooled outcome (new RR: 0.57, 95% CI: 0.39 to 0.86). The results of the subgroup analysis are presented in Table 2. Importantly, the separate analysis of RCTs indicated a significant association between routine ultrasound mapping and lower primary arteriovenous fistula failure rates (RR: 0.37, 95% CI: 0.25 to 0.54). Study location, sample size, year of publication and risk of bias did not significantly influence the overall outcome. The certainty of evidence was evaluated as low due to downgrading in the domains of study limitations and inconsistency.

**Table 2:** Outcomes of the subgroup analysis

Subgroup	Early AVF failure	12-months primary patency
Overall	**0.56 (0.37–0.84)**	**1.33 (1.19–1.47)**
Study location		
Europe	0.59 (0.32–1.08)	**1.35 (1.12–1.63)**
Asia	0.57 (0.30–1.09)	**1.22 (1.12–1.31)**
North America	–	**1.55 (1.32–1.82)**
South America	**0.33 (0.17–0.67)**	–
*P* value for subgroup effect	0.757	0.351
Sample size		
≤ 150 patients	**0.46 (0.24–0.88)**	1.22 (0.99–1.50)
> 150 patients	0.62 (0.37–1.06)	**1.34 (1.19–1.51)**
*P* value for subgroup effect	0.468	0.590
Year of publication		
≤ 2010	**0.45 (0.32–0.64)**	**1.33 (1.14–1.55)**
> 2010	0.75 (0.40–1.39)	**1.33 (1.16–1.52)**
*P* value for subgroup effect	0.091	0.964
Study design		
Cohort study	0.83 (0.50–1.38)	**1.34 (1.19–1.51)**
Randomized controlled trial	**0.37 (0.25–0.54)**	**1.26 (1.02–1.56)**
*P* value for subgroup effect	**0.013**	0.744
Risk of bias		
Low	0.62 (0.27–1.40)	1.15 (0.99–1.35)
Moderate	**0.49 (0.28–0.86)**	**1.31 (1.18–1.46)**
Serious	0.69 (0.36–1.31)	**1.48 (1.00–2.19)**
*P* value for subgroup effect	0.783	0.412

Routine ultrasound examination is compared to physical examination (reference group). Data presented as risk ratio (95% confidence intervals). Bold text indicates statistical significance

*AVF* arteriovenous fistula

### One-year primary arteriovenous fistula patency

**Fig. 3:**
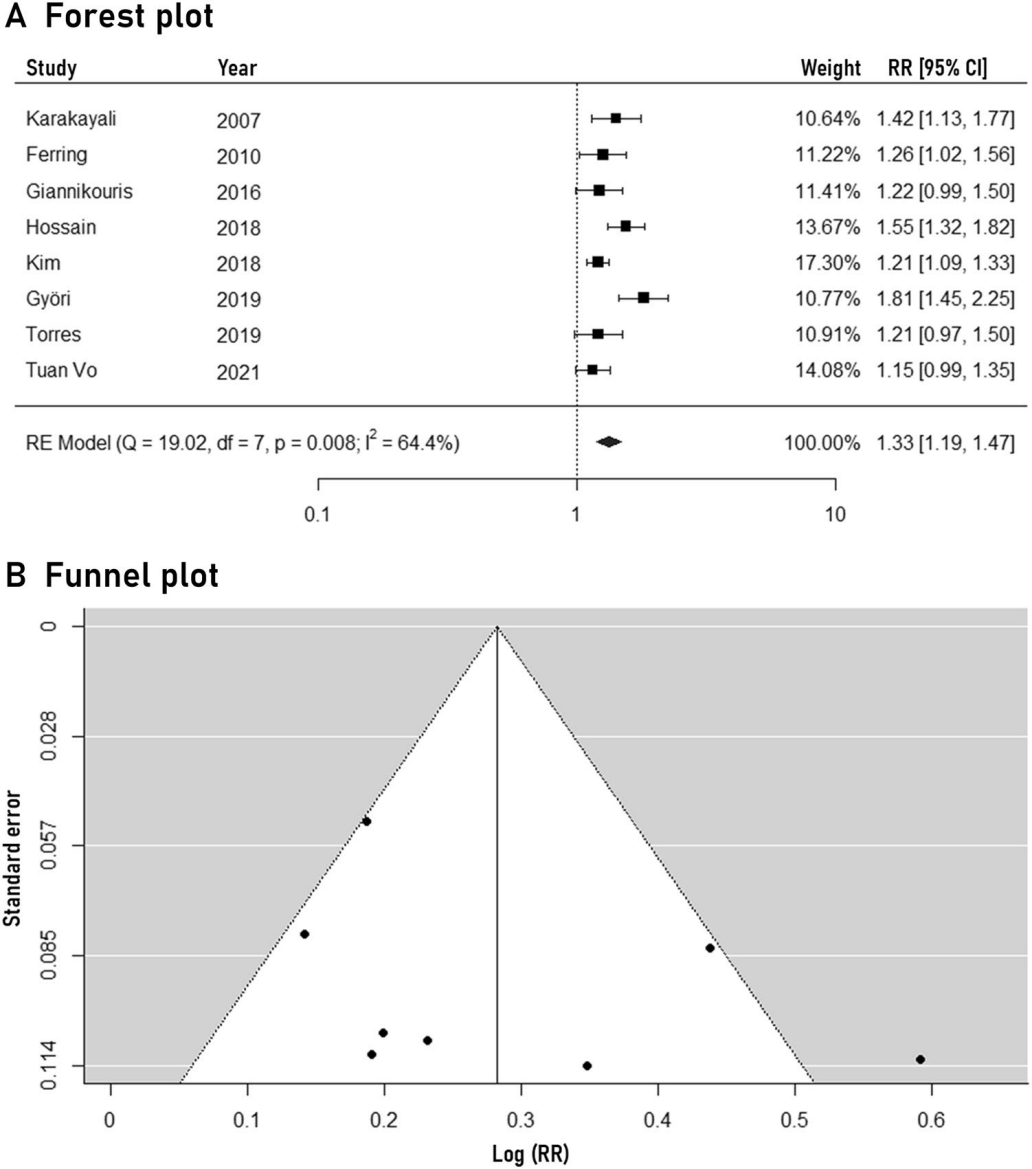
Forest plot (**A**) and funnel plot (**B**) of the pre-operative ultrasound mapping effects on 1-year primary arteriovenous fistula patency

The outcomes of the meta-analysis regarding the primary arteriovenous fistula patency at 12 months are illustrated in Fig. 3. Routine ultrasound mapping was associated with significantly higher rates of 1-year primary arteriovenous fistula patency (RR: 1.33, 95% CI: 1.19–1.47, 8 studies). Statistical heterogeneity was moderate (*I*^*2*^: 64.4%). The 95% predictive intervals remained significant (1.03–1.71). No apparent funnel plot asymmetry was recognized and the trim-fill method identified no missing studies. The subgroup analysis revealed no significant heterogeneity based on study location, sample size, year of publication, design and risk of bias (Table 2). The certainty of evidence was evaluated to be moderate, due to downgrading only in the domain of study limitations.

## Discussion

By including eighteen studies with a total of 3655 patients, the present meta-analysis provides an updated evaluation of both early and one-year patency rates of arteriovenous fistulas created following routine pre-operative ultrasound mapping versus selective ultrasound or physical examination alone. Routine ultrasound examination was associated with significantly better outcomes in terms of early arteriovenous fistula failure, which was even more pronounced when only RCTs were included in the analysis. In addition, the implementation of routine pre-operative mapping was linked to better long-term outcomes, as reflected by the significantly higher rates of 1-year primary patency.

The findings of the present study are in contrast with the results of a previous meta-analysis which concluded that there was no statistically significant difference in favor of pre-operative ultrasound examination. However, the meta-analysis by Wong et al. included only three RCTs reporting only the early maturation rates, and information on the long-term events was lacking, highlighting the need for larger, long-term trials in order to provide more robust results [41]. This consideration was shared by a subsequent meta-analysis by Kosa et al. that concluded that there was no benefit of pre-operative mapping on fistula outcomes compared to physical examination alone [42]. The meta-analysis, however, was based on studies with a high risk of bias which, in combination with the reported low pooled sample size, make these results inconclusive.

Conversely, a meta-analysis by Georgiades et al. [43] highlighted a reduced immediate failure rate in the routine ultrasound group, with the odds of immediate failure being almost three times less. Still, the study did not report on the long-term outcomes and also did not include the results of the RCT by Lopez et al*.* which enrolled 228 patients and reported a significantly higher primary failure rate in the group which underwent clinical examination only (13.6%) compared to the group assessed by ultrasound (4.4%) [23]. Overall, these results indicate the need for routine sonographic evaluation of the veins in both arms to select the best candidate vessels. These benefits have been further confirmed in populations in which physical examination usually fails to identify the best candidate vessels, such as obese patients [6].

The present study has several strengths. A comprehensive literature search was conducted by screening 4 databases without date restrictions. Both RCTs and cohort studies were evaluated separately and together. Extensive subgroup analysis was performed, aiming to explore the potential sources of inter-study heterogeneity, while the calculation of the 95% predictive intervals provided an assessment of the effects to be expected by future studies. Furthermore, the certainty of outcomes was critically appraised following the standardized GRADE approach.

On the other hand, this systematic review and meta-analysis has limitations. Firstly, the analysis was conducted with study-level data and not patient-level data in order to adjust for baseline demographic characteristics that could contribute to confounding bias. Also, the observational studies that were included were rated as harboring a high risk for bias and introduced significant heterogeneity when pooled together with the randomized controlled trials, which had different inclusion criteria and assessed different primary outcomes. It is likewise important to consider that the reporting of access outcomes in hemodialysis trials is very heterogeneous, with limited patient-reported outcomes and great need for standardized outcome measures. In this context, a systematic review by Viecelli et al*.* identified 168 relevant trials with a total of 1426 access-related outcome measures [44]. Access function was assessed in 489 different ways including “mean access blood flow (mL/min)” and “number of thromboses” being used in 27% and 22% of the trials, respectively. Of note, the authors reported that a very limited number of trials included patient-related outcomes with pain and quality-of-life being reported in only 11% and 3% of the studies, respectively. Lastly, the inclusion of patients from different countries and socioeconomic backgrounds may contribute to the high heterogeneity of the pooled estimates and uncertainty for the generalization of the meta-analysis results.

Overall, the findings of this meta-analysis point toward better arteriovenous fistula patency rates with the use of routine pre-operative ultrasound vessel mapping. However, clinicians should be aware of the low to moderate certainty of the existing evidence, mainly due to inter-study heterogeneity resulting from different definitions of endpoints. To this end, future research should focus on the implementation of standardized outcome measures in order to promote consistency and improve the relevance of trial evidence. Specifically, several outcome measures have been suggested, such as uninterrupted arteriovenous fistula use or the ability to receive 2-needle canulation without the need for intervention. In addition, it is important for future studies to include patient-reported outcomes, aiming to take into account quality of life, as well as patients’ preferences and values [45]. Various dialysis-specific questionnaires have been developed to establish patient-reported outcome measures, such as the Kidney Disease Questionnaire (KDQ) [46] and the Hemodialysis Access-Related Quality-of-life (HARQ) tools [47]. It is also important to conduct cost-effectiveness analyses aiming for a reliable economic evaluation of access-related costs, including the need for hospitalizations and surgical procedures.

## Conclusions

The present meta-analysis indicates that the implementation of routine pre-operative ultrasound mapping in addition to physical examination may be associated with beneficial outcomes in terms of both primary failure and 1-year primary patency of arteriovenous fistulas. This effect was apparent even when randomized controlled trials were separately pooled, while the overall credibility of evidence was evaluated to be low to moderate. Further research is needed to confirm these outcomes, as well as to elucidate whether a multidisciplinary approach to vascular access creation involving the routine use of ultrasound is able to affect long-term patency rates, reduce health costs related to access failure complications and improve patients’ quality of life.

### Supplementary Information

Below is the link to the electronic supplementary material.

Supplementary file1 (DOCX 425 KB)
