# Supplementary material for: Pre-operative ultrasound mapping before arteriovenous fistula formation: an updated systematic review and meta-analysis
Source: J Nephrol. 2023 Dec 22;37(2):281–92. doi: 10.1007/s40620-023-01814-6 (PMC11043143; doi:10.1007/s40620-023-01814-6)

**Suppl. Table 1.** Outcome of the ROBINS-I tool for the evaluation of cohort studies

| ***Study*** | ***Bias due to confounding*** | ***Bias in selection of participants into the study*** | ***Bias in classification of exposures*** | ***Bias due to deviations from intended exposures*** | ***Bias due to missing data*** | ***Bias in measurement of outcomes*** | ***Bias in selection of the reported result*** | ***Overall bias*** |
| --- | --- | --- | --- | --- | --- | --- | --- | --- |
| ***2007; Karakayali*** | Moderate | Moderate | Low | Low | Low | Low | Low | Moderate |
| ***2010; Kakkos*** | Serious | Low | Low | Moderate | Low | Low | Moderate | Serious |
| ***2013; Ilhan*** | Moderate | Low | Low | Moderate | Low | Low | Low | Moderate |
| ***2016; Kim*** | Moderate | Moderate | Low | Moderate | Low | Low | Low | Moderate |
| ***2016; Giannikouris*** | Moderate | Moderate | Low | Low | No Information | No Information | Moderate | Moderate |
| ***2016; Mat Said*** | Moderate | Moderate | Low | Low | Low | Low | Low | Moderate |
| ***2016; Martinez*** | Moderate | Low | Low | Low | Low | Low | Low | Moderate |
| ***2018; Hossain*** | Moderate | Moderate | Low | Low | Moderate | Low | Moderate | Moderate |
| ***2018; Kim*** | Moderate | Low | Low | Low | Low | Low | Moderate | Moderate |
| ***2019; Györi*** | Serious | Serious | Low | Low | Low | Moderate | Low | Serious |
| ***2019; Torres*** | Serious | Moderate | Low | Low | Moderate | Low | Low | Serious |
| ***2022; Tuan Vo*** | Low | Low | Low | Low | Low | Low | Low | Low |

**Suppl. Figure 1.** Outcome of the ROB-2 tool for the evaluation of Randomized Controlled Trials


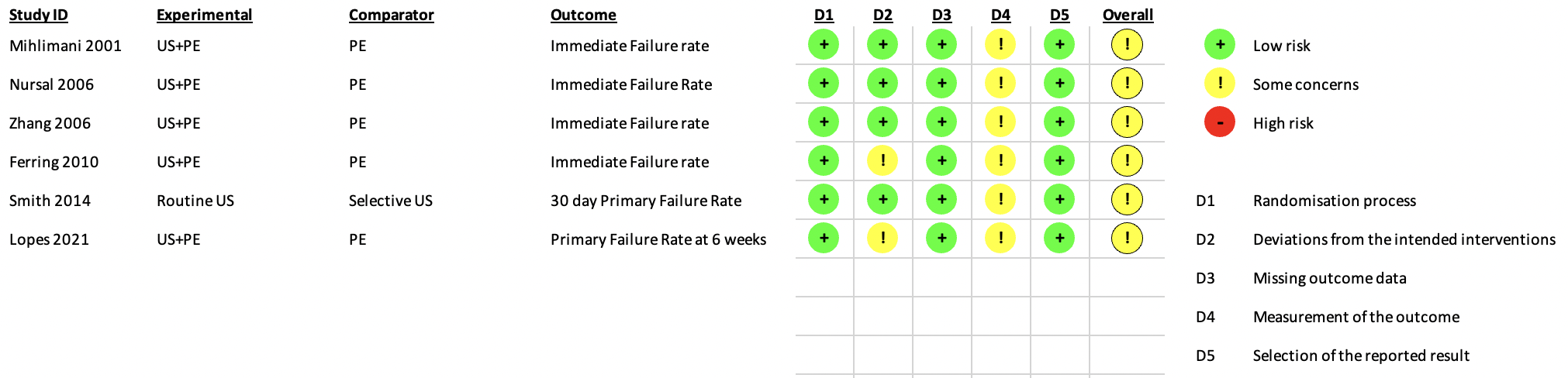

Supplement: Supplementary file 1 — Supplementary file1 (DOCX 425 KB) [file 40620_2023_1814_MOESM1_ESM.docx]
